# Manifest type B Wolff‐Parkinson‐White syndrome complicated with slow/fast atrioventricular nodal reentrant tachycardia: A case report

**DOI:** 10.1002/joa3.70069

**Published:** 2025-04-15

**Authors:** Daiki Yamashita, Yoshihiko Kagawa, Shinichi Harada, Fumiya Uchida, Kaoru Dohi

**Affiliations:** ^1^ Department of Cardiology and Nephrology Mie University Graduate School of Medicine Tsu Japan; ^2^ Ultrasound Examination Center Mie University Hospital Tsu Japan

**Keywords:** AVNRT with bystander atrioventricular AP, catheter ablation, electrophysiologic study, manifest type B Wolff‐Parkinson‐White syndrome, slow/fast atrioventricular nodal reentrant tachycardia

## Abstract

The unstable left‐sided AP was a bystander in AVRT via the right‐sided AP, and the right‐sided AP was a bystander in AVNRT in this case. Interestingly, the right‐sided AP was either part of the circuit or a bystander.

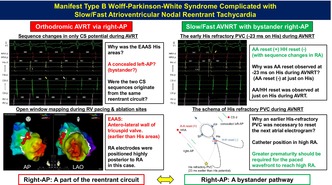

Wolff‐Parkinson‐White (WPW) syndrome is a congenital syndrome that affects approximately 0.1% of the population.^1^ Atrioventricular nodal reentrant tachycardia (AVNRT) is the most common regular supraventricular tachycardia (SVT) and is dependent on dual atrioventricular (AV) nodal pathway physiology for the propagation of its reentry circuit.^2^ Here, we report a case of type B WPW syndrome complicated by slow/fast AVNRT.

A 44‐year‐old woman with a history of WPW syndrome complained of palpitations. Physical examination revealed a heart rate of 70 bpm. Electrocardiogram (ECG) revealed type B WPW syndrome (Figure 1A). During electrophysiologic study (EPS), a right atrium‐coronary sinus (RA‐CS) catheter was placed into the RA and CS, and two quadripolar catheters were positioned in the right ventricle (RV) and His bundle areas (Figure 1B). At baseline, the sinus cycle length was 750 ms. The atrio‐His (AH) interval was 50 ms, and the His‐ventricular (HV) interval was −14 ms. The His bundle areas were the retrograde earliest atrial activation site (EAAS) during RV pacing, and two CS sequence patterns were observed (proximal‐to‐distal and distal‐to‐proximal). Dual AV nodal physiology and decremental property of AV conduction were not observed during programmed pacing then. The effective refractory period of the antegrade accessory pathway (AP) was 300 ms, the retrograde AV nodal pathway was 300 ms, and the retrograde AP was 320 ms. Para Hisian pacing showed a fusion/fusion pattern.

**FIGURE 1:**
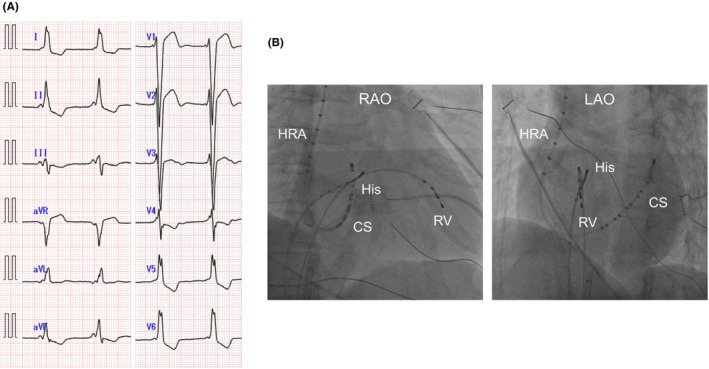
(A) ECG showed manifest type B WPW syndrome. We estimated the localization of the AP to the anterior wall or antero‐lateral wall of the tricuspid valve with reference to the Arruda algorithm. (B) Catheter position in the EPS. AP, accessory pathway; CS, coronary sinus; ECG, electrocardiogram; EPS, electrophysiologic study; HRA, high right atrium; LAO, left anterior oblique position; RAO, right anterior oblique position; RV, right ventricle; WPW, Wolff‐Parkinson‐White.

The narrow QRS tachycardia was induced during programmed stimulation from RA in the setting of isoproterenol (ISP) loading. Tachycardia cycle length (TCL) was 385 ms. The activation sequence of the His bundle electrode was H‐V‐A. The retrograde EAAS was His bundle areas. The activation sequence of the CS changed from distal‐to‐proximal (SVT1) to proximal‐to‐distal (SVT2) 30 s later, without changing the TCL and the activation sequence of RA, His, and RV electrodes (Figure 2A). In SVT2, His refractory premature ventricular contractions (PVC) at the same time as His potential reset both A–A interval and H–H interval sequentially. V–A–V activation sequence, correct postpacing interval‐tachycardia cycle length (cPPI‐TCL) ≤110 ms, and orthodromic His capture were observed with ventricular overdrive pacing (VOP). Therefore, SVT2 was diagnosed as orthodromic atrioventricular reentrant tachycardia (AVRT).

**FIGURE 2:**
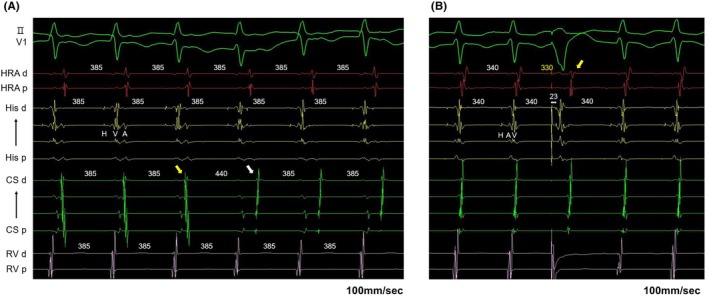
(A) TCL was 385 ms. The activation sequence of the His bundle electrode was H–V–A pattern during tachycardia. The retrograde EAAS was His bundle areas during SVT. The activation sequence of the CS changed from distal‐to‐proximal (SVT1; yellow arrow) to proximal‐to‐distal (SVT2; white arrow) from 30 s later, without changing of the TCL and the activation sequence of RA, His, and RV electrodes. (B) His refractory PVC at 23 ms earlier than His potential reset A–A interval with sequence changes in atria but did not reset H–H interval. Yellow arrow showed sequence changes in atria due to early atrial capture via right‐sided AP in SVT3. A–A, Atrio–Atrio; AP, accessory pathway; CS, coronary sinus; EAAS, earliest atrial activation site; H–H, His–His; PVC, premature ventricular contraction; RA, right atrium; RV, right ventricle; SVT, supraventricular tachycardia; TCL, tachycardia cycle length.

Another narrow QRS tachycardia (SVT3) was also induced during programmed stimulation from RA. The TCL was 340 ms. The activation sequence of the His bundle electrode was H‐A‐V. The interval of HA was <70 ms, and AH was >200 ms. The retrograde EAAS was His bundle areas. His refractory PVC at the same time as His potential didn't reset both A‐A interval and H‐H interval sequentially. However, His refractory PVC at 23 ms earlier than His potential reset A‐A interval with sequence changes in atria but didn't reset H‐H interval (Figure 2B). V‐A‐V activation sequence, cPPI‐TCL >110 ms, and antidromic His capture were observed with VOP. Moreover, the atrial sequence transitioned from partial RA entrainment to whole atrial entrainment during VOP. Therefore, SVT3 was diagnosed as slow/fast AVNRT bystander atrioventricular AP.

First, the AP was targeted. A three‐dimensional electroanatomical mapping system (CARTO 3; Biosense Webster, USA) was used, open window mapping (OWM) in the RA‐RV was performed using a multi‐electrode catheter (Octaray; Biosense Webster, USA) during RV pacing (Figure 3A). The AP potential was automatically annotated on the antero‐lateral wall of the tricuspid valve in the early‐meets‐late gap. Radiofrequency (RF) ablation was performed, and the anterograde conduction of the AP disappeared. After AP ablation, AH jump appeared during programmed pacing, and slow/fast AVNRT appeared with AH jump. Thereafter, slow pathway ablation was performed. VA conduction via only the AV node was observed in the setting of ISP loading. No tachycardia was induced; AH jump disappeared. The patient exhibited no recurrence of arrhythmia since then.

**FIGURE 3:**
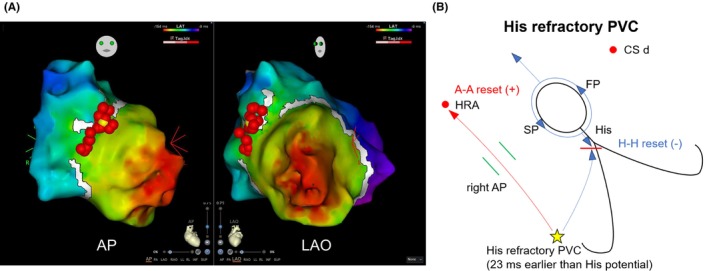
(A) Open window mapping in the RA–RV was performed using a multielectrode catheter (Octaray; Biosense Webster) during RV pacing. The AP potential was automatically annotated on the antero‐lateral wall of the tricuspid valve in the early‐meets‐late gap (yellow tag; successful ablation site, Red tags; total ablation sites). (B) The mechanism of the findings in Figure 2(B) is shown in the schematic. A–A, Atrio–Atrio; AP, accessory pathway; AP, Anterior Posterior position; CS, coronary sinus; FP, fast pathway; H–H = His–His; LAO, left anterior oblique position; RA, right atrium; RV, right ventricle; SP, slow pathway.

Few previous reports have shown the manifest type B WPW syndrome complicated with AVNRT. Moreover, the intermittent eccentric electrical conduction (CS potential) was observed in the present case. A concealed left‐sided AP should be responsible for the distal‐to‐proximal CS activation, as the CS musculature is absent at the position of the distal bipole of the CS catheter. However, because of its intermittent electrical conduction properties, a detailed evaluation could not be performed in SVT1. Moreover, we did not use adenosine during RV pacing after ablation to disclose the concealed left‐sided AP.

AVNRT with a bystander atrioventricular AP pattern is interesting and makes an accurate SVT diagnosis more difficult. Nagashima et al. reported a systematic method for diagnosing AVNRT with a bystander concealed nodoventricular pathway (cNVP).^3^ In SVT3, His refractory PVC occurred 23 ms earlier than His potential reset A–A interval with sequence changes in the atria but did not reset the H–H interval. Sequence changes in the atria were observed due to early atrial capture via the right‐sided AP (Figure 3B). This finding is rare and indicates AVNRT with a bystander atrioventricular AP pattern.^4^

However, His refractory PVC at the same time during AVNRT as His potential did not reset both A–A interval and H–H interval sequentially in this case. We speculate that the cause of this finding was the catheter position in the RA. The RA electrodes were positioned highly posterior to the RA and far from the tricuspid valve annulus. Therefore, the retrograde EAAS was His bundle areas during RV pacing and SVT2. Moreover, His refractory PVC at the same time as His potential during AVRT reset both A–A interval and H–H interval sequentially. It supports our explanation of why an earlier His‐refractory PVC was necessary during AVNRT to reset the next atrial electrogram. The RA was activated by the wavefront via the fast pathway but not via the AP during AVNRT, so greater prematurity should be required for the paced wavefront to reach the high RA.

In conclusion, we encountered a rare case of type B WPW syndrome complicated by AVNRT. The unstable left‐sided AP was a bystander in AVRT via the right‐sided AP, and the right‐sided AP was a bystander in AVNRT in this case. Interestingly, the right‐sided AP was either part of the circuit or a bystander.

## CONFLICT OF INTEREST STATEMENT

Authors declare no conflict of interests for this article.

## INFORMED CONSENT

Written informed consent was obtained from the patient.
